# Uncommon presentation of isolated laryngeal sarcoidosis in a young man

**DOI:** 10.1016/j.amsu.2022.104216

**Published:** 2022-07-31

**Authors:** Bayan O. Besharah, Revan A. Mujahed, Haddad H. AlKaf, Sherif K. Abdelmonim, Ali Aboloyoun Mohamed, Hasheema Alsulami, Mohammad A. Al-Essa

**Affiliations:** a ORL-H&N Resident PGY5, Head, Neck and Skull Base Health Center, King Abdullah Medical City Hospital, Mecca, 21955, Saudi Arabia; b Medical Intern, Umm Al-Qura University, Mecca, 21955, Saudi Arabia; c Head and Neck Surgical Oncology, Head, Neck and Skull Base Health Center, King Abdullah Medical City Hospital, Mecca, 21955, Saudi Arabia; d Ain Shams University, Cairo, Egypt; e Assiut University, Egypt; f King Abdullah Medical City Hospital, Mecca, 21955, Saudi Arabia

## Abstract

• Sarcoidosis is a chronic multisystemic granulomatous disease with unknown etiology.
• Sarcoidosis affects the head and neck region in only 10%–15% of cases.
• Larynx with turban-like thickening should raise high suspicions of laryngeal sarcoidosis.
• Sarcoidosis is diagnosed after laboratory, endoscopic, and histopathology findings exclude other differentials.
• Isolated laryngeal sarcoidosis is rare, making early diagnosis challenging and rising misdiagnosis.

A 26-year-old male without any known prior medical issues experienced a sore throat that evolved into dyspnea after receiving oral antibiotics three years prior to presentation at our institution. Due to airway compromise, he underwent surgical tracheostomy as well as tonsillectomy, as the initial diagnosis was complicated tonsillitis. The patient was then referred to our hospital as his symptoms did not improve. Microlaryngeal surgery (MLS) examination and biopsy with debulking of the base of the tongue lesion were performed with successful decannulation of the tracheostomy which done by consultant of head and neck surgeon at our center. Histopathological examination was non diagnostic for any disease. The patient missed his postoperative appointment and was lost to follow-up for two years until he presented again with complaints of dyspnea, globus sensation, and dysphagia, which progressed over time. There was no history of constitutional symptoms, dysphonia, or stridor, nor any history of smoking, alcohol use, recent travel, or contact with patients with respiratory illness. Flexible laryngoscopic examination showed polypoidal changes over the epiglottis, arytenoids, and aryepiglottic folds with moderate pale-pink mucosal edema. No abnormality was detected in the glottic and subglottic areas (Fig. 1).

**Fig. 1:**
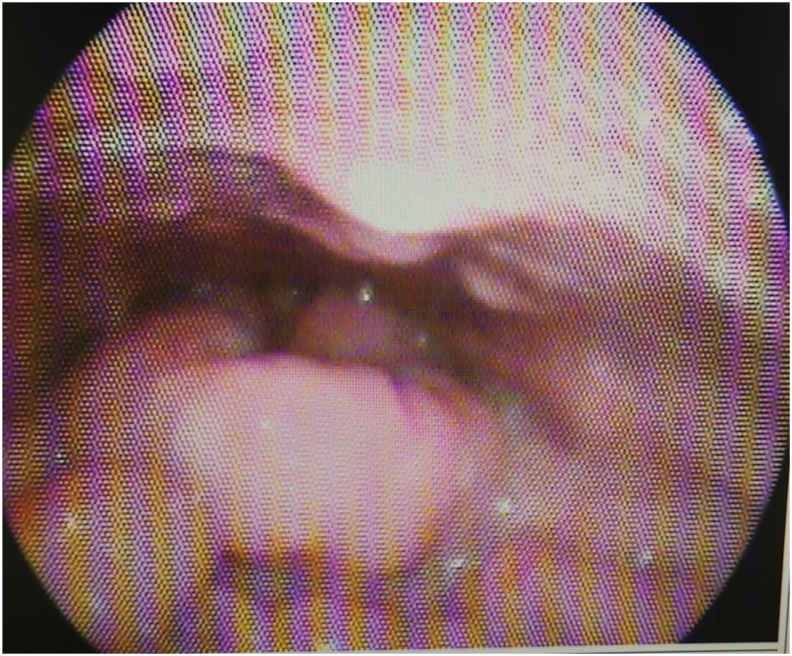
Flexible laryngoscopic view shows polypoidal changes over the epiglottis, arytenoids, and aryepiglottic folds with moderate pale-pink mucosal edema. (For interpretation of the references to colour in this figure legend, the reader is referred to the Web version of this article.)

We admitted the patient after discussion with him the option of the management and the need for further workup, MLS for biopsy with debulking, and tracheostomy. In addition, blood samples were sent for angiotensin-converting enzyme level, mycobacterium tuberculosis culture and polymerase chain reaction, rheumatoid factor level, *human immunodeficiency virus testing*, *c and p* antineutrophil cytoplasmic antibodies, anti nuclear antibodies, and venereal disease research laboratory test which, which were all within normal limits. The patient was positive, however, for Epstein Barr Virus (EBV) IgG. Neck computed tomography (CT) revealed a lobulated soft tissue lesion identified in the lingual surface of the epiglottis measuring approximately 2.6 × 1.5 cm with nodular thickening of the epiglottic and aryepiglottic folds causing moderate airway narrowing.

The results of histopathological biopsies showed no clear phenotypic evidence of aberrant lymphocytes in flow cytometry but had evidence of chronic granulomatous inflammation and occasional multinucleated giant cells. An immunohistochemistry study showed negative EBV.

Further serological workup (Quantiferon test) and CT Chest were sent to rule out tuberculosis and pulmonary sarcoidosis showed a negative result for both. The patient's final diagnosis was isolated laryngeal sarcoidosis. He was started on systemic steroids and followed up for 24 months with significant improvement and successful weaning from tracheostomy (Fig. 2) [1].

**Fig. 2:**
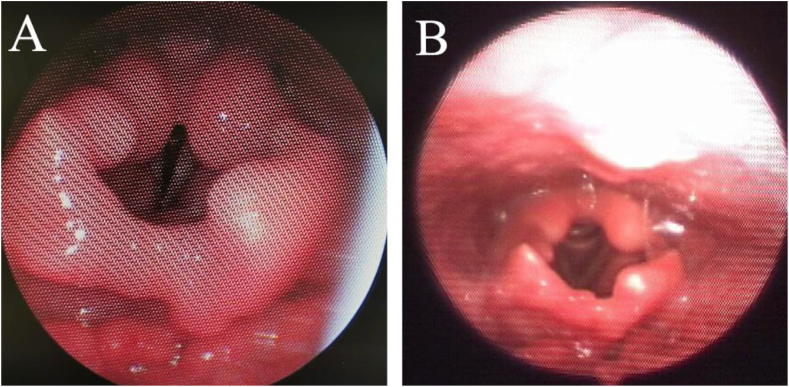
Flexible laryngoscopic view: (A) After three months postoperative shows significant improvement of the mucosa of epiglottis, arytenoids, and aryepiglottic folds. (B) After 24 months of starting oral steroids, mucosal edema greatly improved.

## 1 What is your diagnosis?

A. Sarcoidosis,
B. EBV,
C. TB,
D. Lymphoma.

## 2 Diagnosis

A. Sarcoidosis.

## 3 Discussion

Supraglottitis is inflammation of supraglottic structures that may lead to significant airway obstruction if treatment is delayed [2]. Infection is the most common etiology, but other differentials that vary from traumatic injury, granulomatosis with polyangiitis, and sarcoidosis should be investigated [2].

Sarcoidosis is a chronic multisystemic granulomatous disease with unknown etiology that commonly affects the lungs, eyes, and lymph nodes [3]. Furthermore, the disease commonly presents in middle-age at the third to the fourth decade of life with a female predominance [4,5]. However, sarcoidosis affects the head and neck region in only 10%–15% of cases [4]. Laryngeal involvement accounts for 0.5%–5% of sarcoid patients, while isolated laryngeal sarcoid is very uncommon [2,4]. Patients usually present with dysphagia, dyspnea, pharyngeal globus, dysphonia, and dry cough. In severe or complicated cases due to delayed diagnosis, patients can present with a life-threatening condition in which the upper airway becomes completely obstructed, and tracheostomy may be needed. This occurs in 15% of the cases [6,7]. Flexible laryngoscopic examination usually shows pale and edematous mucosa mainly involving the epiglottis as the most common site with a characteristic turban-like appearance, which could be related to rich lymphatic supply to this tissue, followed by involvement of the arytenoids and aryepiglottic folds [4]. Involvement of the glottic and subglottic areas is less common, and vocal cord mobility could be affected in approximately 24% of cases either due to sarcoid infiltration of the larynx or direct neural involvement of the disease or by the compressive effect of lymphadenopathy [7,8].

The diagnosis of sarcoidosis is made after excluding other differentials by laboratory and histological examination in conjunction with endoscopic findings and histopathological confirmation of noncaseating granulomatous inflammation [2,7]. Computed tomography and positron emission tomography (CT/PET) aid in defining the specific active laryngeal site in sarcoidosis with 85% sensitivity [7]. Direct laryngoscopic examination under general anesthesia is mandatory with multiple biopsies to rule out malignancy and other possible diagnoses [5]. In the case presented above, all the laboratory workups were unremarkable except for EBV-IgG, which was ruled out as a causative factor by histopathological examination.

However, due to limited data, treatment options for laryngeal sarcoidosis are still under investigation [4]. Systemic steroids are the first-line therapy, and prednisone (40–60 mg/day in adults) is the most favorable medication for a total period of 6–12 months [7]. Cytotoxic medications such as methotrexate, cyclosporine, hydroxychloroquine, and azathioprine are an option to be used in patients who have recalcitrant symptoms or for whom to long-term steroid use is contraindicated [3,7]. Other alternative systemic options that showed efficacy in laryngeal sarcoidosis are biological agents targeting tumor necrosis factor [3,5,9]. In addition, Kelleher KJ et al. suggested starting with sirolimus (rapamycin) and other mechanistic targets of rapamycin (mTOR) inhibitors in recalcitrant cases [3]. Local treatment is still an option providing less morbidity risk, either via local steroid injection or surgical debulking by cold instruments, carbon dioxide (CO2) lasers, or microdebriders, especially in severely symptomatic cases with a well-circumscribed lesion [2,5].

In conclusion, isolated laryngeal sarcoidosis is a rare disease, which makes early diagnosis more challenging and increases the chance of misdiagnosis.

## Ethical approval

IRB.

Waive has no deviation from standard care was done.

## Sources of funding

None.

## Author contribution

The authors confirm contribution to the paper as follows: study concept and design: Mohammad A Al-Essa; data collection: Revan A Mujahed, Ali Aboloyoun Mohamed; writing the paper: Bayan O Besharah; data interpretation and manuscript review: Haddad H AlKaf, Sherif K Abdelmonim, Hasheema Alsulami; principal investigator and senior editor: Mohammad A Al-Essa. All authors reviewed the results and approved the final version of the manuscript.

## Consent

Written informed consent was obtained from the patient for publication of this case report and accompanying images. A copy of the written consent is available for review by the Editor-in-Chief of this journal on request.

## Registration of research studies

1. Name of the registry:
2. Unique Identifying number or registration ID:
3. Hyperlink to your specific registration (must be publicly accessible and will be checked):

## Guarantor

Alessa, mohammad Ali.

Head and neck oncology and Microvascular reconstruction Surgeon.

King Abdullah Medical city Hospital.

Alessa.mohammad@gmail.com.

## Declaration of competing interest

None.
